# Membrane Transition Temperature Determines Cisplatin Response

**DOI:** 10.1371/journal.pone.0140925

**Published:** 2015-10-20

**Authors:** Krishnan Raghunathan, Aarif Ahsan, Dipankar Ray, Mukesh K. Nyati, Sarah L. Veatch

**Affiliations:** 1 Department of Biophysics, University of Michigan, Ann Arbor, Michigan, United States of America; 2 Department of Radiation Oncology, University of Michigan, Ann Arbor, Michigan, United States of America; Institut Jacque Monod, Centre National de la Recherche Scientifique, FRANCE

## Abstract

Cisplatin is a classical chemotherapeutic agent used in treating several forms of cancer including head and neck. However, cells develop resistance to the drug in some patients through a range of mechanisms, some of which are poorly understood. Using isolated plasma membrane vesicles as a model system, we present evidence suggesting that cisplatin induced resistance may be due to certain changes in the bio-physical properties of plasma membranes. Giant plasma membrane vesicles (GPMVs) isolated from cortical cytoskeleton exhibit a miscibility transition between a single liquid phase at high temperature and two distinct coexisting liquid phases at low temperature. The temperature at which this transition occurs is hypothesized to reflect the magnitude of membrane heterogeneity at physiological temperature. We find that addition of cisplatin to vesicles isolated from cisplatin-sensitive cells result in a lowering of this miscibility transition temperature, whereas in cisplatin-resistant cells such treatment does not affect the transition temperature. To explore if this is a cause or consequence of cisplatin resistance, we tested if addition of cisplatin in combination with agents that modulate GPMV transition temperatures can affect cisplatin sensitivity. We found that cells become more sensitive to cisplatin when isopropanol, an agent that lowers GPMV transition temperature, was combined with cisplatin. Conversely, cells became resistant to cisplatin when added in combination with menthol that raises GPMV transition temperatures. These data suggest that changes in plasma membrane heterogeneity augments or suppresses signaling events initiated in the plasma membranes that can determine response to cisplatin. We postulate that desired perturbations of membrane heterogeneity could provide an effective therapeutic strategy to overcome cisplatin resistance for certain patients.

## Introduction

Cisplatin is a highly effective chemotherapeutic agent and has been successfully used in the treatment of several types of tumors. Cisplatin exerts its cytotoxicity primarily by crosslinking DNA, which in turn interferes with DNA transcription, replication, and triggers a DNA damage response leading to cell-death [1]. However, with time, many patients acquire resistance to cisplatin through distinct mechanisms [2]. One mechanism employed by cancer cells is reduction of the intracellular concentration of the drug through active up-regulation of efflux pumps such as P-glycoprotein [3] and copper transporters [4,5]. Some cancer cells increase their ability to repair DNA damage [6], or alter or bypass the DNA damage response signals that typically trigger apoptosis [7] and thereby acquire drug resistance. Also, changes in several off-target signaling pathways are implicated in the development of cisplatin resistance, including those involved in cell growth, differentiation, and stress responses [8,9]. Many cases of acquired resistance involve a combination of multiple mechanisms [2].

While cisplatin primarily targets DNA, many membrane-associated signaling pathways are implicated in cisplatin resistance. For example, cisplatin activates EGFR at the plasma membrane through ligand- and DNA damage response-independent mechanisms [10–12]. The degradation of EGFR upon cisplatin treatment is also linked to cell survival [8]. In addition, cisplatin has been shown to interact directly with specific plasma membrane lipids [13–16], and numerous past studies have correlated changes in membrane fluidity with cisplatin action and resistance [17–20]. For example, a relationship has been demonstrated between membrane fluidity and resistance to cisplatin by measuring the anisotropy of membrane probes [21]. Cisplatin has also been found to increase plasma membrane fluidity in HT29 cells as measured by EPR order parameter and this increase in fluidity is correlated with clustering of apoptotic receptors [22]. Previous studies have also shown plasma membrane composition to be different between cisplatin -sensitive and -resistant cell lines [19,23]. For instance, incorporating heptadecanoic acid to cells has been observed to increase membrane fluidity and increase cisplatin resistance [19]. Interestingly, changes in membrane lipid composition upon cisplatin treatment are also different between resistant and sensitive cells [24]. It has been argued that increased membrane fluidity favors apoptosis and therefore cisplatin sensitivity by facilitating the clustering of death receptors such as FAS at the plasma membrane, possibly by modulating ‘lipid rafts’ [21,25,26]. Overall, physical properties of plasma membrane lipids show intriguing correlations with the sensitivity of cells to cisplatin, possibly by modulating growth factor and/or apoptosis signaling cascades. As previous studies have implicated lipid heterogeneity in both apoptotic and growth pathways [27–31], we hypothesize that at least some aspects of cisplatin sensitivity could have its origins in the mixing properties of plasma membrane lipids.

Our understanding of how plasma membrane lipids can influence the functional organization of proteins at the cell surface has vastly improved over the last decade [32–35]. The plasma membrane of mammalian cells can support two distinct liquid phases with different average protein and lipid compositions, called liquid-ordered and liquid-disordered phases. These coexisting liquid phases are directly visible when plasma membranes are isolated from cortical cytoskeleton and viewed at reduced temperature [36], or when indirectly probed by isolating detergent resistant membranes using sucrose gradients at low temperature [37,38]. It is widely thought that phase-related structures also persist at physiological temperature in intact cells as small and dynamic domains, and that these domains play important roles in regulating a range of cellular processes [39–43]. One proposed physical basis for ‘raft’ heterogeneity is that structure arises because cell plasma membranes have compositions close to a miscibility critical point at growth temperature [44,45], which is a special thermodynamic condition where extended regions of differing composition can form spontaneously at equilibrium. Within this model, the size, composition, and stability of domains is dependent on the distance of the membrane from the critical point, both in composition and temperature. Previous studies have identified several compounds that modulate transition temperatures in both purified membranes and isolated plasma membrane vesicles [28,46–48]. These compounds are predicted to modulate the magnitude of heterogeneity in intact cell membranes at fixed growth temperatures in a way that could impact cellular functions.

Here, we investigate the relationship between chemoresistance to cisplatin and the effect that cisplatin has on giant plasma membrane vesicles (GPMVs) isolated from the same cell type. We show that cells that are more sensitive to cisplatin produce GPMVs whose miscibility transition temperatures are more greatly affected by incubation with cisplatin. Further, we provide evidence that this relationship is causal, by demonstrating that cisplatin resistance can be altered in the presence of agents that modulate transition temperatures. Finally, we show that modulation in chemoresistance is not due to increased cisplatin concentration within the cell.

## Materials and Methods

### Cell culture

UMSCC1, UMSCC11B, UMSCC17B, and ME180-pt cell lines were provided by Dr. Thomas E. Carey (University of Michigan, Ann Arbor, MI). The UMSCC cell lines were initially characterized by Dr. Carey's lab as a part of University of Michigan Squamous Cell Carcinoma cell lines. The genotype and origins of these cell lines are listed on the UM Head and Neck SPORE Tissue Core website[49]. Cells were grown in RPMI 1640 media with Penn/strep and 10% fetal bovine serum. RBL-2H3 cells [50] were a kind gift of Barbara Baird and David Holowka (Cornell University, Ithaca, NY) and grown in MEM media with 20% FBS and 0.1% Gentamycin.

#### Preparation of giant plasma membrane vesicles (GPMVs)

GPMVs were prepared as described previously [46]. In brief, the cells were first washed with a GPMV buffer of 2 mM CaCl_2_ /10 mM Hepes/0.15 M NaCl, pH 7.4, then labeled with 200 μg/ml DiI-C_12_ and 1% methanol in the same buffer for 10min at 37°C. Cells were then washed and incubated in the GPMV buffer with the addition of 25 mM formaldehyde and 2 mM DTT for up to three hours at 37°C with gentle agitation. GPMVs are shed into the buffer which is decanted prior to imaging.

#### Transition temperature measurements

Transition temperatures were obtained by quantifying the fraction of GPMVs containing coexisting liquid phases as a function of temperature using a fluorescence microscopy assay that has been described previously [36,46]. Briefly, DiI-C_12_ labeled GPMVs were placed between two coverslips, mounted on a home-built temperature stage, and imaged using an inverted microscope (IX81; Olympus, Center Valley, PA) with a 40X 0.95 NA air objective and an Neo SCMOS camera (Andor, South Windsor, CT). Images of fields of vesicles were acquired over a range of fixed temperatures. In post processing, vesicles were identified as containing either a single or coexisting phases by visual inspection, and counting was accomplished using custom software written in Matlab. The fraction of vesicles containing two coexisting phases as a function of temperature was fit to a sigmoid function to obtain the transition midpoint.

#### Cell counting

10^5^ cells were plated and allowed to adhere overnight. The complete media was then replaced with serum free RPMI media and in some cases supplemented with additional compounds, as specified. After 24 hours, detached cells were removed through rinsing, adherent cells were lifted by addition of trypsin-EDTA (0.25%, Gibco) then counted using a Coulter particle count and size analyzer. In all cases, cell counts for treated cells were normalized by a control sample grown for 24h in serum free media without additional treatments.

The half maximal inhibitory concentration (IC_50_) values for cisplatin were determined by fitting normalized cell count data (R) acquired over a range of cisplatin concentrations (C) to Equation:

$$R=IC_{50}{}^{m}/\left(IC_{50}{}^{m}+C^{m}\right)$$ (1)

where m is a fit parameter that specifies the slope of the curve.

#### Immunoblotting

5×10^5^ cells were plated for each condition and grown overnight. The media was then replaced with serum free RPMI media with or without additional treatments and grown for 24 hours, then the cultures washed to remove cell debris. Adherent cells were scrapped into PBS containing sodium orthovanadate and protease inhibitor mixture (Roche Diagnostic, Co) pelleted at 13000 RPM for two minutes then suspended in Laemmli buffer (63 mmol/L Tris-HCl, 2% (w/v) SDS,10% (v/v) glycerol, and 0.005% (w/v) bromphenol blue) containing 100 mM NaF, 1 mM Na_3_VO_4_, 1mM PMSF, and 1 μg/ml aprotinin. Samples were lysed through sonication then centrifuged at 13000 RPM for 15 min at 4°C. The supernatant was removed and heated to 95°C and 20–40 μg of total protein per well was electrophoresed on a 4% to 12% bis-tris precast gel (Invitrogen). Total protein was assayed using Bradford assay. Gels were transferred onto a polyvinylidene difluoride membrane then incubated in a TBS blocking buffer (137 mmol/L NaCl, 20 mmol/L Tris-HCl (pH 7.6), 0.1% (v/v) Tween 20, 3% BSA and 1% normal goat serum). Membranes were incubated overnight at 4°C in the corresponding primary antibody, washed, and then incubated for 1hr in a horseradish peroxidase-conjugated secondary antibody (Cell Signaling). After washes, the westerns were developed using enhanced chemiluminescence plus reagent (Amersham Biosciences)

#### Inductive coupled plasma-optical emission spectroscopy (ICP-OES)

5×10^6^ cells were grown for each condition overnight, transferred to a serum free media with the corresponding treatment for 24 hours, then rinsed to remove non-adherent cell debris. Adherent cells were trypsinized and counted for later normalization. Cells were pelleted then lysed in 500ul concentrated nitric acid overnight. 2.5ml of additional nitric acid was added prior to platinum detection via ICP-OES using an Perkin-Elmer Optima 2000 DV (Perkin-Elmer, Wellesley, MA) equipment. Yttrium was used as an internal standard with a detection wavelength of 371.029 nm. Platinum was detected at the well separated peak of 214.423 nm, and calibrated using samples containing known concentrations of cisplatin. The detected levels of cisplatin were normalized to the cell count to produce units of molecules of cisplatin per cell.

We then estimate the predicted cisplatin levels inside cells by rearranging Eq 1. To do this, we first assume that the average number of cisplatin molecules within cells (N) is proportional to the external concentration. Internal concentration can then be calculated using the fraction of cells that survive a cisplatin treatment (R) according to Eq 2,

$$N=k{\left[\left(1-R\right)/R\right]}^{1/m},$$ (2)

where m is determined by fitting dose response curves to obtain m = 0.43 and k is determined from data acquired for 10μM cisplatin in UMCC1 cells in the absence of additional treatments (N = 1.44±0.3, R = 0.61±0.03) to obtain k = 4.1±0.75. Error in k is obtained by propagating errors in N and R through Eq 2. Values are predicted by plugging measured R values under the given conditions into Eq 2, and error bounds are obtained by again propagating through the calculation.

### Statistical analysis

Data are represented as the mean ± the standard error of the mean (SEM). For pair-wise statistical analysis of significance a student t-test was used. P<0.05 was deemed as significant. For samples with more than two conditions, a one way Anova was used, and results were deemed significant when F_crit_ < F.

## Results

### Cisplatin lowers critical temperatures in RBL-2H3 derived GPMVs

**Fig 1:**
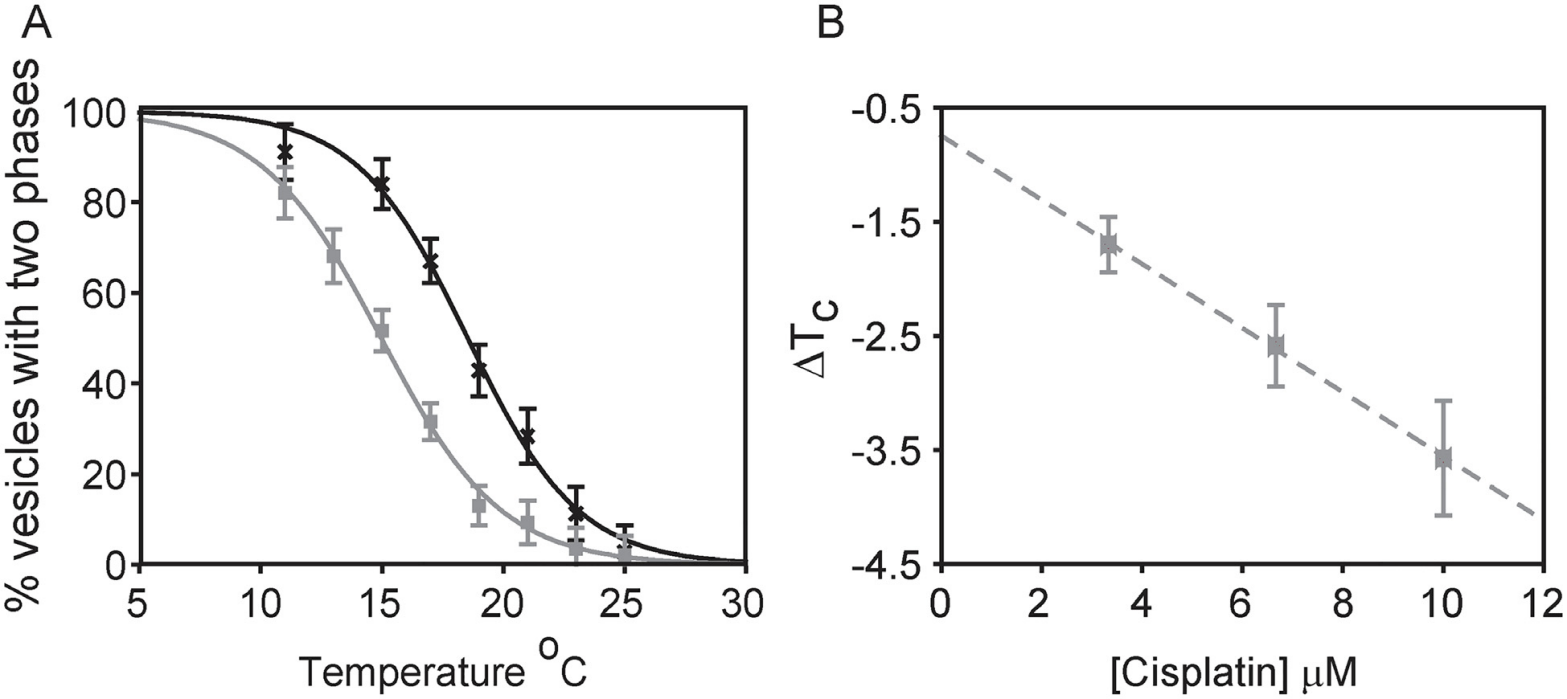
Cisplatin lowers transition temperatures in GPMVs isolated from RBL cells. (A) The fraction of GPMVs with coexisting liquid phases as a function of temperature for RBL-2H3 derived GPMVs imaged in the absence (black crosses) or presence (grey squares) of 10μM cisplatin in a representative measurement. Data points are fit to a sigmoid function to determine the temperature where half of vesicles contain coexisting liquid phases, which is the average transition temperature (T_C_). (B) Measurements like that shown in A were repeated to obtain ΔT_C_ over a range of cisplatin concentrations. Points represent an average of three independent trials, and error bars represent the SEM. The dotted line is meant only as a visual guide.

Individual plasma membrane vesicles from RBL-2H3 cells separate into coexisting liquid-ordered and liquid-disordered phases at low temperatures [44]. Vesicles are in a single liquid phase at elevated temperatures and undergo micron-scale fluctuations at temperatures within several degrees of the miscibility transition [51], which is frequently close to room temperature when GPMVs are prepared using the reducing agent DTT [44]. Individual vesicles prepared from cells plated in the same dish display large variations in transition temperatures [36]. Therefore, we quantified average transition temperatures by measuring the fraction of vesicles that contain coexisting liquid phases as a function of temperature [46] as described in Methods and shown in Fig 1A. The average transition temperature is defined as the temperature where 50% of vesicles contain coexisting phases.

Incubating RBL-2H3 derived GPMVs with cisplatin acts to shift average transition temperatures to lower values, as can be seen in the representative measurement of Fig 1A. In this example, freshly prepared GPMVs had an average transition temperature of 18.6°C in the absence of cisplatin and this average transition temperature reduced to 15.0°C when the same preparation of vesicles was examined in the presence of 10μM cisplatin. We find that absolute transition temperatures of control and treated GPMVs were variable when this measurement was repeated using GPMVs isolated from different preparations of RBL-2H3 cells, but the transition temperature decrease upon addition of 10μM cisplatin remained constant within experimental errors. This observation is consistent with past work characterizing effects of n-alcohols on transition temperatures [46]. For this reason, we report the change in transition temperature, ΔT_C_, rather than absolute values. ΔT_C_ varied linearly with concentration of cisplatin over a clinically relevant range of concentrations (Fig 1B).

### ΔT_C_ correlates with cisplatin sensitivity in four different cancer cell lines

**Fig 2:**
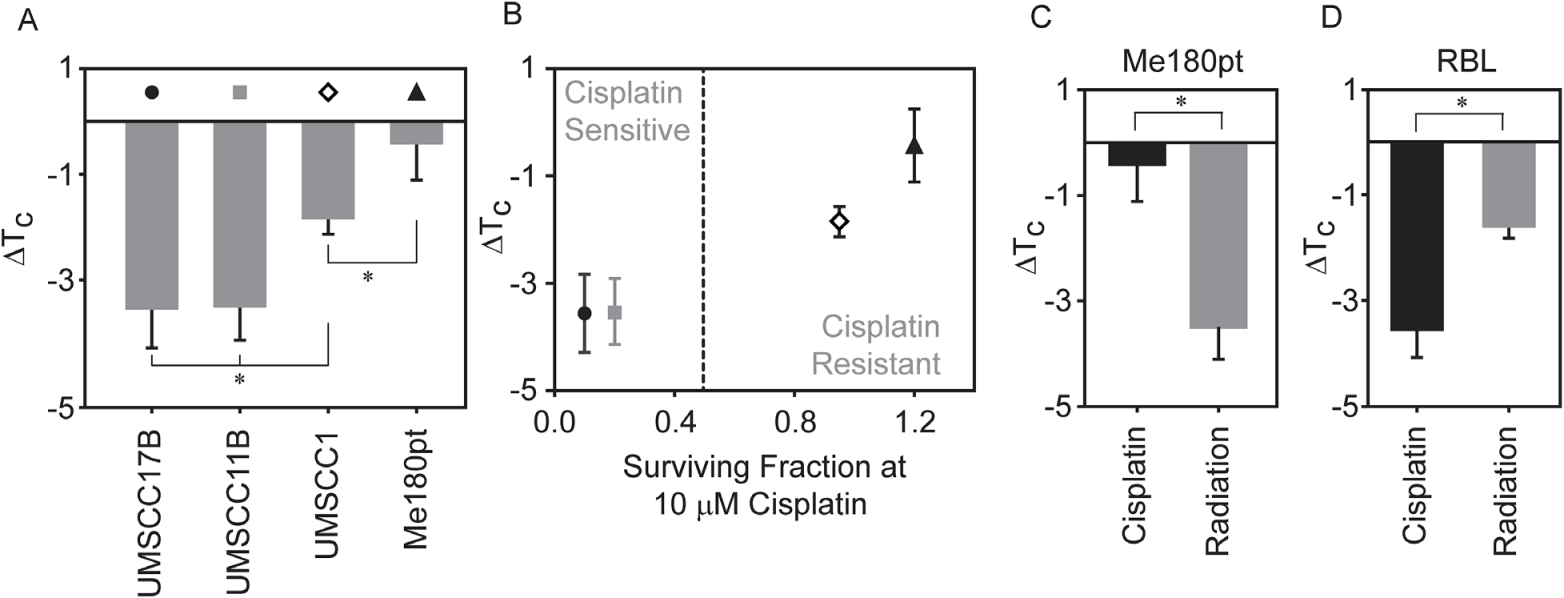
Changes in transition temperature in GPMVs correlate with the cell lines resistance to cisplatin. (A) GPMVs were isolated from four cell-lines as described in the Methods section. The transition temperature shifts are reported by comparing the transition temperatures of GPMVs probed in the presence of 10 μM cisplatin to untreated GPMVs. (B) Data points in panel A were plotted against a previously reported measure of surviving fraction to cisplatin obtained using clonogenic assays for the same four cell lines [8]. Surviving fraction was measured using a clonogenic survival assay. Surviving fractions were measured 72 hours after treatment with 10uM cisplatin. The straight line is drawn to visually distinguish sensitive and resistant celllines. Transition temperature shifts upon incubation with 10 μM cisplatin or exposure to 10 Gy irradiation for GPMVs isolated from ME-180 pt cells (C) and RBL cells (D). In all cases, points represent the average of at least 3 independent measurements and error bounds represent the standard error of the mean. Significance between transition temperature shift measurements were evaluated using t-tests.

While RBL-2H3 derived GPMVs have been used widely in biophysical studies to study lipid heterogeneity, this cell line is not commonly used to explore cisplatin resistance. To better address the relevance of our findings, we prepared GPMVs from three head and neck cancer cell lines (UMSCC1, UMSCC11B and UMSCC17B) and one cervical squamous cell carcinoma cell line (ME-180Pt). These four cell lines were chosen because they display a range of response to cisplatin when tested for clonogenic survival with UMSCC10B and UMSCC11B being the most sensitive to cisplatin while Me180Pt being the most resistant to it [8]. We observed a range of ΔT_C_ shifts when GPMVs were isolated from these cell types and treated with 10μM cisplatin (Fig 2A). Cisplatin treatment led to a downward shift in transition temperature for UMSCC17B and UMSCC11B derived GPMVs (-3.6± 0.7°C and -3.5±0.6°C respectively), similar to that observed in RBL-2H3. Transition temperatures of UMSCC1 GPMVs also shifted downward, but to a lesser extent upon cisplatin treatment (ΔT_C_ = -1.85±0.3°C), and no significant shift was observed in case of ME-180Pt (-0.43±0.7°C).

Interestingly, we find that the magnitude of ΔT_C_ upon cisplatin treatment of isolated GPMVs correlates with the previously reported response of intact cells to this drug [8], as seen in Fig 2B. UMSCC17B and UMSCC11B cells are most sensitive to cisplatin treatment and also produce GPMVs whose transition temperature is depressed by more than 3°C in the presence of 10 μM cisplatin compared to untreated vesicles. Me180pt cells are resistant to this dose of cisplatin, and correspondingly, we did not observe a significant ΔT_C_ upon cisplatin treatment of GPMVs produced from this cell line. The UMSCC1 cell line showed both intermediate cisplatin sensitivity and an intermediate ΔT_C_ in our GPMV measurement.

To explore if this transition temperature lowering effect was specific to cisplatin treatment, or if it might be generalized to other types of cancer treatments, we measured the effects of ionizing radiation on transition temperature on GPMVs isolated from the cisplatin resistant cell line, ME-180Pt. Previous studies have shown that radiation altered the fluidity of both artificial and cellular membranes [52–55]. We find that GPMVs isolated from Me-180pt cells show a downward shift in transition temperature when exposed to 10 Gy irradiation (ΔT_C_ = -3.5±0.6°C) as compared to control vesicles while downward shift in transition temperature was distinctly absent upon cisplatin treatment. Contrastingly, in the case of GPMVs isolated from RBL cells, both treatment with identical doses of radiation or cisplatin produced a measurable decrease in transition temperature compared to an untreated control (Fig 2D). Differences in the transition temperature changes between radiation and cisplatin treated samples is probably indicative of a celllines' response to its corresponding treatment. This result supports the possibility that changes in membrane transition temperatures could be a general theme influencing the sensitivity to these treatments.

### Biochemical modulators of T_C_ alter cisplatin sensitivity

**Fig 3:**
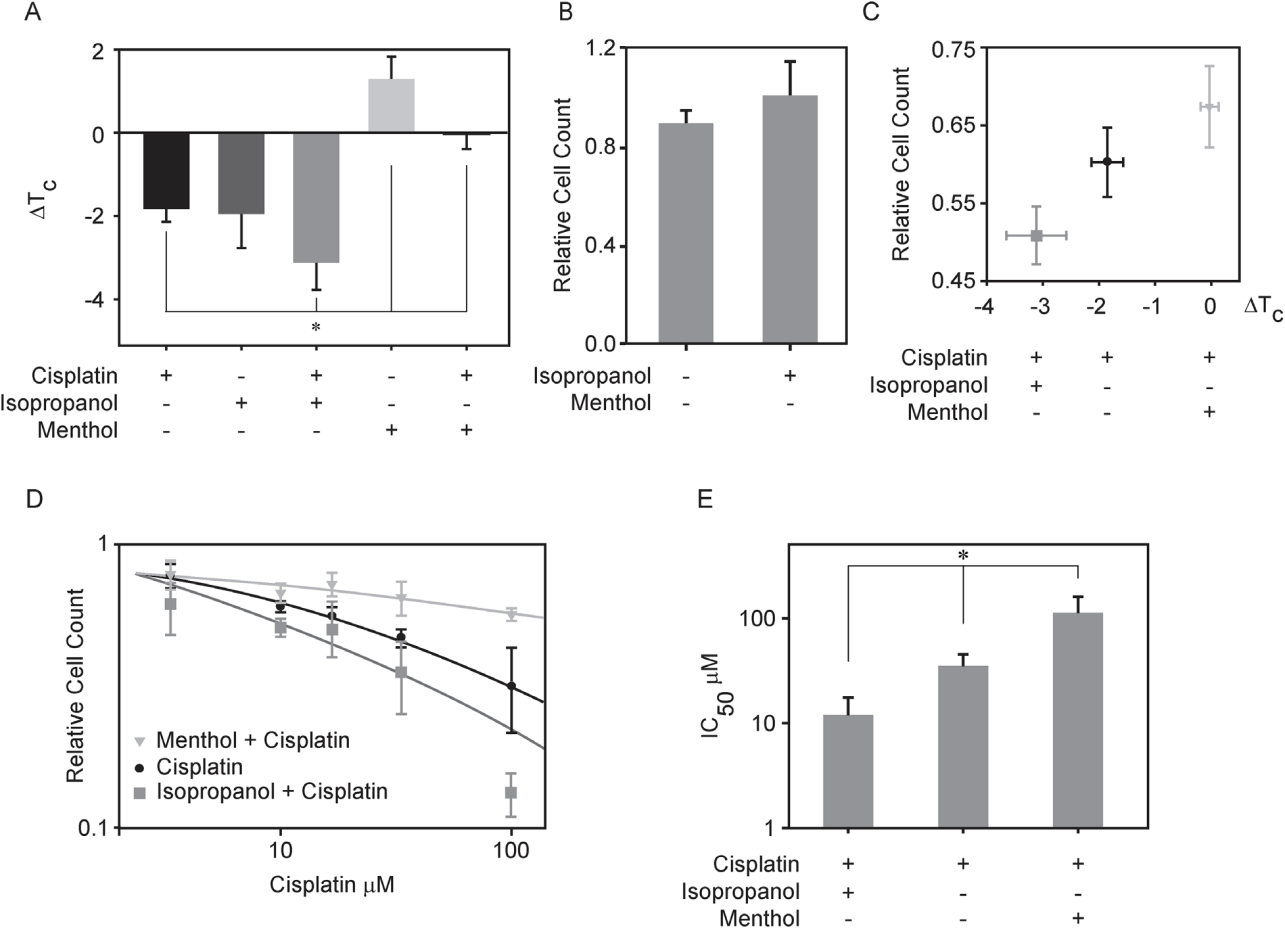
Modulating transition temperature affects cisplatin mediated cellular response in UMSCC1 cells. (A) Transition temperature shifts measured for GPMVs isolated from UMSCC1 in the presence of 50mM isopropanol or 100 μM menthol or each of these treatments in combination with 10μM cisplatin. (B) Efficacy of 50mM isopropanol and 100μM menthol action on UMSCC1 cells calculated as the number of cells present after 24h of treatment divided by the number of cells present in an untreated control. (C) Efficacy of cisplatin action as a function of the transition temperature shift effected by the treatment in isolated GPMVs shown in A. Efficacy of cisplatin action on UMSCC1 cells as above was computed as above in (B) by dividing the number of cells present after 24h of treatment compared to the number of cells present in an untreated control. (D) Plots show relative cell counts as a function of cisplatin concentration either in the presence or absence of 50mM isopropanol or 100μM menthol. Each point represents the average and SEM of at least 4 independent measurements, and lines are fit to Eq 1. (E) Average IC_50_ values as determined by fitting Eq 1 to individual dose response curves. Values represent an average and SEM over at least 4 independent measurements.

In order to explore if changes in plasma membrane transition temperatures are upstream of cisplatin sensitivity, we measured cisplatin sensitivity in the presence of additional reagents that can shift transition temperatures in isolated GPMVs (Fig 3A). UMSCC1 cells were selected for this study because they exhibit intermediate sensitivity to cisplatin. We have previously shown that isopropanol lowers transition temperatures in RBL-2H3 derived GPMVs [46] and here we find that 50mM isopropanol lowers T_C_ by 1.9 ±0.8°C in UMSCC1 derived vesicles. When 50mM isopropanol is added in combination with 10μM cisplatin, the effect on ΔT_C_ is roughly additive, with an aggregate ΔT_C_ of -3.1±0.5°C (Fig 3A). This shift was comparable to the ΔT_C_ measured for cisplatin alone in the sensitive cell lines investigated (UMSCC17B). Menthol is a hydrophobic compound that partitions into membranes and activates cold sensitive transient receptor potential cation channel subfamily M member 8 (TRPM8) channels [56]. 100μM menthol raised critical temperatures in UMSCC1 derived GPMVs by +1.3±0.5°C. Adding 100μM menthol in combination with 10μM cisplatin to isolated vesicles acts to cancel the T_C_ modulating effects of both compounds (0.0±0.36°C). This lack of shift in T_C_ was comparable to the effect of cisplatin alone on GPMVs isolated from the cell line investigated that is the most resistant to this drug (Me-180Pt).

In addition to characterizing the effects of compounds on isolated GPMVs, we also assayed cisplatin sensitivity in intact cells under similar conditions by comparing the number of adherent cells present following a 24h incubation with the specified treatments normalized to the number of adherent cells present in an untreated control (Fig 3C). We counted almost 20% fewer UMSCC1 cells when were treated with a combination of cisplatin and isopropanol when compared to cells treated with cisplatin alone, indicating that isopropanol increases the sensitivity of UMSCC1 cells to this drug. In contrast, 11% more cells were counted in samples treated with menthol and cisplatin compared to treatment by cisplatin alone, indicating that the presence of menthol protected these cells from the toxic effects of cisplatin. No significant change in cell population was observed when cells were incubated with either isopropanol or menthol in the absence of cisplatin when compared to untreated control (Fig 3B).

We determined the effective cisplatin IC_50_ by counting cells over a range of cisplatin concentrations in the presence of a fixed concentration of either isopropanol or menthol (Fig 3D). We found that the IC_50_ values of different agents varied quite dramatically. While the IC_50_ was 36 μM for cisplatin in UMSCC1, this reduced to 12 μM when cells were incubated with cisplatin in combination with 50mM isopropanol (Fig 3E). Conversely, the IC_50_ of cisplatin increased to 115 μM when cells were treated with cisplatin in combination with 100 μM menthol. Taken together, these results indicate that cisplatin sensitivity can be altered by incubating with additional compounds that modulate ΔT_C_.

**Fig 4:**
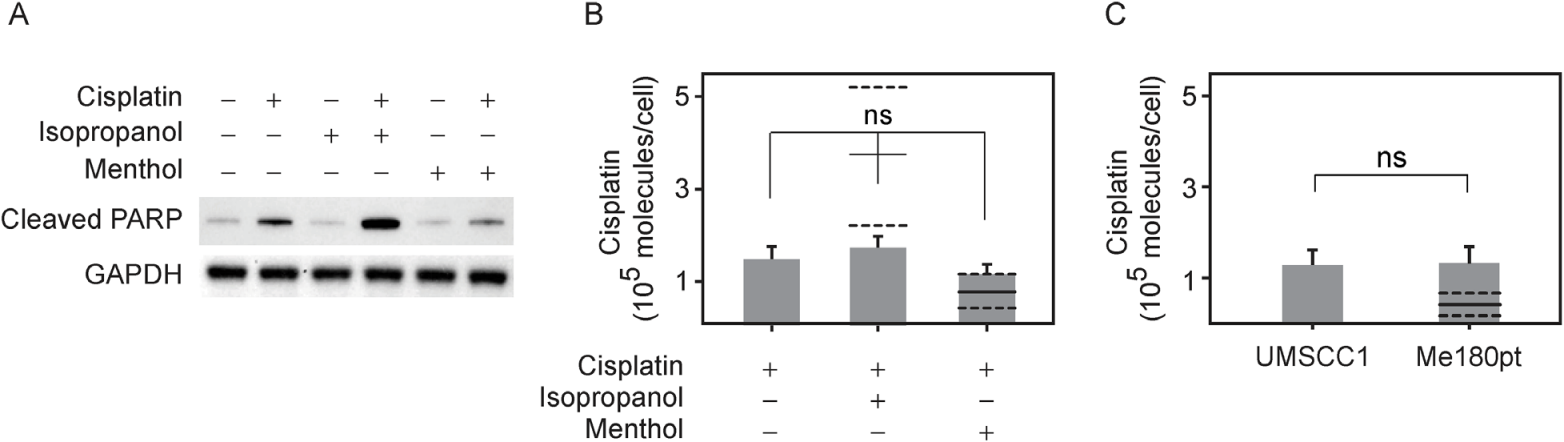
Co-incubation of cisplatin with isopropanol leads to enhanced apoptosis without an increase in intercellular cisplatin concentration. (A) Expression levels of cleaved PARP, an apoptotic marker, as measured by western blot for cells incubated with 50mM isopropanol plus 10μM cisplatin, with 100μM menthol plus 10μM cisplatin or with 10μM cisplatin alone along with cisplatin free controls (B) Levels of intracellular cisplatin were measured using optical emission spectrometry for the three treatments, 50mM isopropanol plus 10μM cisplatin, with 100μM menthol plus 10μM cisplatin or with 10μM cisplatin alone. The differences between the three treatments are not statistically significant (n = 8 trials). Also shown are predicted levels of cisplatin obtained by assuming that intracellular cisplatin that is directly proportional to the external concentration determines the extent of cell death (as described in Methods). The solid line denotes the predicted mean theoretical value and corresponding dashed lines denote error bounds. (C) Levels of intracellular cisplatin for UMSCC1 and the more resistant cell line Me-180pt treated with 10μM cisplatin. The solid line as described previously denotes predicted levels given the assumptions stated in 4B and dashed lines indicate error bounds.

We further correlated this cell count result by measuring apoptosis upon these different treatments. Previous work has shown that cisplatin induces apoptosis in sensitive cells within 12 hrs [57,58]. A well characterized biochemical indicator of the onset of apoptosis is the cleaved product of poly (ADP-ribose) polymerase (PARP), and we probed for the presence of cleaved PARP through Western blot (Fig 4A). Consistent with our cell counting results, cleaved PARP levels were elevated in cells treated with isopropanol and cisplatin compared to cisplatin alone, and reduced in cells treated with menthol and cisplatin. Cleaved PARP levels were similar to control samples in cells treated with either isopropanol or menthol alone, indicating that these compounds alone were not toxic at the concentrations used.

### Intracellular cisplatin levels do not correlate with cisplatin resistance

We then tested the hypothesis that modulating the transition temperature of plasma membrane could lead to increased influx or decreased efflux of cisplatin in the cell. Cells can potentially execute this through increased activity of P-Glycoprotein or other proteins involved in cisplatin transport [2,4,5,59]. To probe this, we directly measured intracellular platinum levels using inductively coupled plasma optical emission spectroscopy (ICP-OES). This has been previously used to determine the absolute levels of cisplatin levels in cells [4,60]. Using this method, we did not observe statistically significant differences in platinum levels in UMSCC1 cells after a 24 hour treatment with either cisplatin alone, or cisplatin in combination with either isopropanol or menthol.

There are several theoretical models [61–63] that relate cell death with drug concentration and with intracellular drug concentrations. We used one of these approaches to theoretically predict the intracellular cisplatin concentration assuming that the number of cisplatin molecules within the cell is simply proportional to its extracellular concentration over a wide range for cells treated with cisplatin alone, and that co-treatment with isopropanol or menthol acts to alter this proportionality constant through actions on efflux pumps. Within this framework, we expect to observe significantly more cisplatin within isopropanol and cisplatin treated cells when compared to cisplatin treatment alone, as shown in Fig 4B. This is inconsistent with the measured value, suggesting that isopropanol does not work through a mechanism of altering efflux pumps alone, as has been observed for other chemosensitizers [64]. This calculation also predicts reduced intracellular cisplatin in cells co-treated with menthol when compared to cells treated with cisplatin alone, but the error bounds are too large to exclude this as a possible mechanism in our measurements. A derivation of this calculation is provided in Methods.

Also, we did not observe significant differences in intracellular cisplatin between ME-180Pt and UMSCC1 cells treated with the drug, although the two cell lines differ in their sensitivity to cisplatin (ref. Fig 4C). These results suggest that at least for these cells and experimental conditions, cisplatin sensitivity is not affected through changes in the transport of cisplatin. Instead, it is likely that changes in sensitivity may be due to other factors already associated with cisplatin resistance, such as up-regulation of DNA repair machinery or changes in plasma membrane that affect the activity of apoptotic receptors.

## Discussion

Plasma membrane has been shown to play a wide role in modulating the effects of several drugs. Previous work from our group showed an interesting relationship between transition temperature modulation by general anesthetics and their anesthetic potencies [46]. Work from the Levental group [28] has shown that bile acids modulate transition temperature of plasma membrane and this in turn affects cellular signaling. Further, Hancock and others [27,65,66] have shown that non-steroidal drugs affect plasma membrane heterogeneity and the Ras nanoclusters in plasma membrane. NSAIDs have also been postulated to decrease the risk of cancer through alterations in Ras nanoclustering via changes in plasma membrane heterogeneity [27]. These studies support a role for plasma membrane transition temperature in modulating diverse cellular responses even in carcinogenesis.

Here, we report an intriguing correlation between the sensitivity of a cell to cisplatin and the magnitude of shifts in the miscibility transition temperature induced by cisplatin in plasma membrane vesicles isolated from the same cells. It has been hypothesized that this transition temperature predicts the magnitude and size of heterogeneity in intact cells, with lower miscibility transition temperatures implying a reduction in membrane heterogeneity at growth temperature. In addition, cisplatin sensitivity can be modulated through biochemical treatments that augment transition temperature shifts, suggesting that the effects on membrane mixing properties are a cause and not a consequence of cisplatin sensitivity. A recent paper has shown cisplatin to interact with the head group of phosphatidylcholine which is enriched in the outer leaflet of the plasma membrane [16]. This interaction is not surprising considering that cisplatin has also been shown to interact with phosphotidylserine head groups which are enriched in the inner membrane [13,15]. We propose that interactions of cisplatin with the plasma membrane act to promote mixing of plasma membrane components, which manifests as reduced transition temperatures in isolated plasma membrane vesicles. Since cisplatin treatment takes membranes further from conditions with a stabilized liquid-ordered phase, it is expected that this would also be correlated with reduced ordering of lipid chains. Thus, this work potentially provides a conceptual framework to interpret the body of existing literature that correlates cisplatin resistance with changes in plasma membrane fluidity.

**Fig 5:**
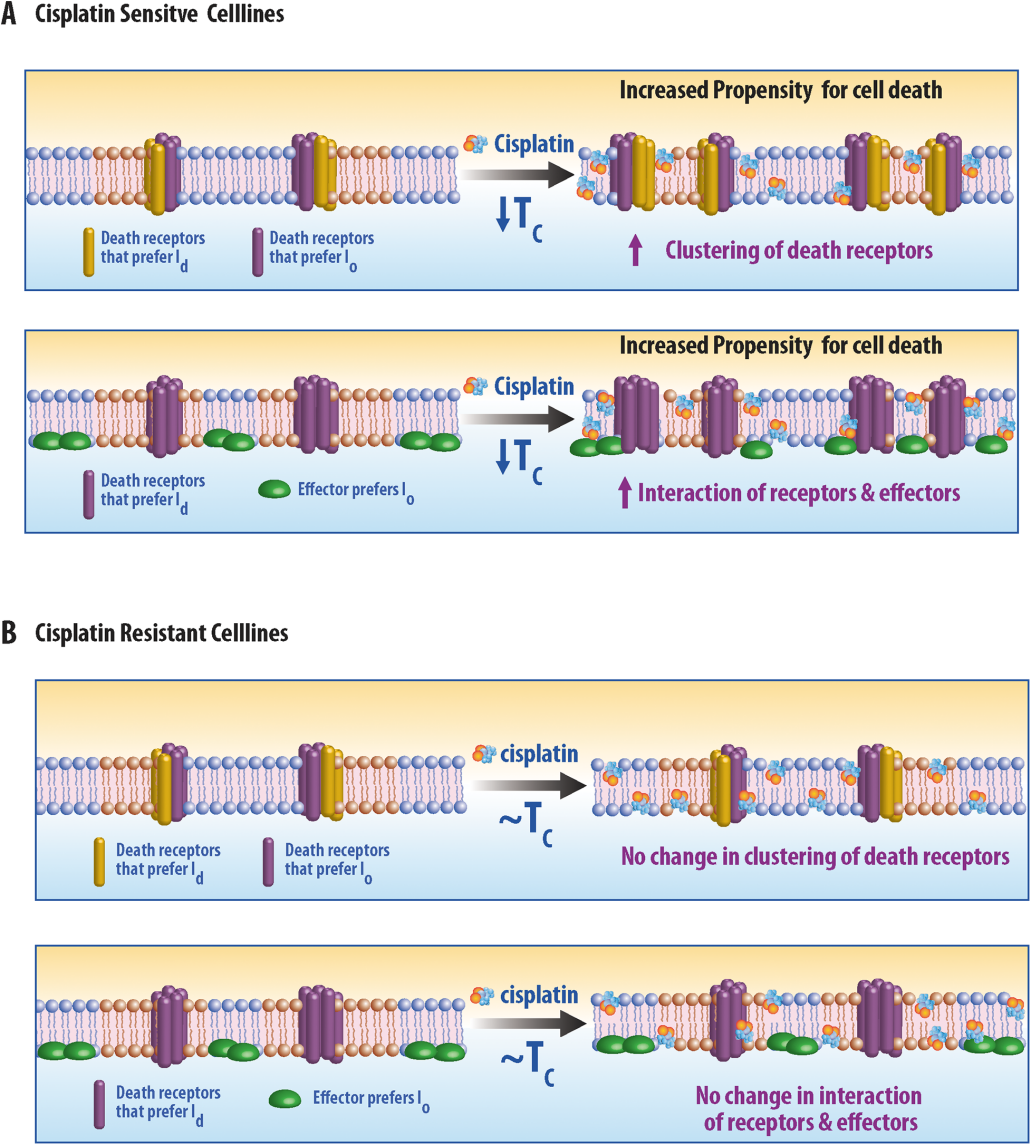
Model for cisplatin mediated activation of death receptors. (A) Response of sensitive cell lines to cisplatin. (Top) Two sets of death receptors, one which prefers the liquid disordered phase (ld) and other that prefers liquid ordered phase (lo). Cisplatin lowers the transition temperature which in turn allows for increased interactions between death receptors. (Bottom) Alternatively, it is possible that an effector molecule of an death receptor, prefers a phase distinct from the phase preference of the receptor. Cisplatin lowers the transition temperature, reducing the size and stability of membrane domains, and increasing the accessibility of the receptor to the effector. (B) Response of a resistant cell line to cisplatin. Interaction of cisplatin with the plasma membrane of resistant cell line do not alter the lipid heterogeneity and hence do not affect activation or response of death receptors.

Our results suggest that a reduction in the magnitude of membrane heterogeneity is causally related to increased sensitivity to cisplatin, although the biochemical mechanism mediating this effect remains unknown. Our data suggest that mechanisms of sensitivity are more likely rooted in the early stages of apoptosis initiation which occur at the plasma membrane rather than in the influx or efflux of cisplatin. Several pathways associated with apoptotic death receptor [67,68] involve clustering of receptors and downstream signaling partners in the plasma membrane, and membrane heterogeneity (e.g. lipid rafts) has been implicated in this signaling cascade. Our result suggests that in certain cells, cisplatin treatment induces death-receptor signaling via destabilization of membrane domains in the plasma membrane. It is plausible that large FAS clusters are more stable under conditions with reduced membrane heterogeneity, or that reduced heterogeneity favors interactions between death receptors and downstream signaling that promote apoptosis [25,69]. Two possible scenarios are illustrated schematically in Fig 5. In the first scenario, two types of death receptors are present, each preferring to localize within different membrane domains (Fig 5a, top). In the second scenario, death receptors and effector molecules partition into different membrane domains (Fig 5a, bottom). In both these cases, decreasing membrane heterogeneity will lead to greater activation of receptors by enabling more frequent contact between proteins. In case of resistant cells such as Me180pt, the interaction of cisplatin with the plasma membrane does not lead to destabilization of membrane heterogeneity (Fig 5b) and hence does not modulate interactions between proteins through this mechanism and hence the response to cisplatin is muted. Interestingly, we have also recently found that plasma membrane transition temperatures are reduced in cells soon after they are treated with the death receptor ligand TRAIL, while transition temperatures are elevated under growth conditions that support rapid cell division[70]. It is possible that suppression of membrane heterogeneity is a general condition that inhibits cellular proliferation and supports apoptotic signaling.

Finally, this work suggests the possibility of developing a novel class of chemosensitzer for cisplatin that targets membrane physical properties. In this work, we used isopropanol to increase cisplatin sensitivity in several cell lines, but we expect that other compounds could produce similar effects, likely with greater potency. It is possible that some previously characterized chemosensitizers such as plant extracts [68], local anesthetics, and nonsteroidal anti-inflammatory drugs [46] could function through this mechanism, as some previous work has shown that some of these compounds also modulate miscibility transition temperatures in model membranes [20,66,68,71]. Further work is needed to probe the clinical relevance of this approach. Drug resistance in cancer cells has multiple origins, and our findings suggest a theoretical foundation for understanding one such mechanism and a novel path for therapeutic intervention.
